# A multimodal imaging study of recognition memory in very preterm born adults

**DOI:** 10.1002/hbm.23405

**Published:** 2016-09-20

**Authors:** Chieh‐En Jane Tseng, Seán Froudist‐Walsh, Philip J. Brittain, Vyacheslav Karolis, Chiara Caldinelli, Jasmin Kroll, Serena J. Counsell, Steven C.R. Williams, Robin M. Murray, Chiara Nosarti

**Affiliations:** ^1^ Department of Psychosis Studies, Institute of Psychiatry, Psychology and Neuroscience King's College London United Kingdom; ^2^ Centre for the Developing Brain, Division of Imaging Sciences and Biomedical Engineering King's College London United Kingdom; ^3^ Centre for Neuroimaging Sciences, Institute of Psychiatry, Psychology and Neuroscience King's College London United Kingdom

**Keywords:** very preterm, recognition memory, tractography, functional MRI

## Abstract

Very preterm (<32 weeks of gestation) birth is associated with structural brain alterations and memory impairments throughout childhood and adolescence. Here, we used functional MRI (fMRI) to study the neuroanatomy of recognition memory in 49 very preterm‐born adults and 50 controls (mean age: 30 years) during completion of a task involving visual encoding and recognition of abstract pictures. T1‐weighted and diffusion‐weighted images were also collected. Bilateral hippocampal volumes were calculated and tractography of the fornix and cingulum was performed and assessed in terms of volume and hindrance modulated orientational anisotropy (HMOA). Online recognition memory task performance, assessed with *A* scores, was poorer in the very preterm compared with the control group. Analysis of fMRI data focused on differences in neural activity between the recognition and encoding trials. Very preterm born adults showed decreased activation in the right middle frontal gyrus and posterior cingulate cortex/precuneus and increased activation in the left inferior frontal gyrus and bilateral lateral occipital cortex (LOC) compared with controls. Hippocampi, fornix and cingulum volume was significantly smaller and fornix HMOA was lower in very preterm adults. Among all the structural and functional brain metrics that showed statistically significant group differences, LOC activation was the best predictor of online task performance (*P* = 0.020). In terms of association between brain function and structure, LOC activation was predicted by fornix HMOA in the preterm group only (*P* = 0.020). These results suggest that neuroanatomical alterations in very preterm born individuals may be underlying their poorer recognition memory performance. *Hum Brain Mapp 38:644–655, 2017*. © **2016 The Authors Human Brain Mapping Published by Wiley Periodicals, Inc.**

## INTRODUCTION

Very preterm birth (<32 weeks of gestation) is associated with structural and functional brain alterations that persist into early adulthood and often correlate with cognitive performance (Bjuland et al., 2013; Mullen et al., 2011; Nosarti et al., 2014; Taylor et al., 2011). Memory is thought to be one of the core components underlying a variety of cognitive tasks (Rose and Feldman, 1996). In particular, recognition memory, defined as the ability to differentiate between new and previously encountered stimuli, is essential for adapting to the environment (Danckert et al., 2007) and is crucial for learning (Riesenhuber and Poggio, 2000).

Memory and learning impairments are often exhibited in preterm survivors (Aanes et al., 2015; Nosarti et al., 2014; Omizzolo et al., 2014; Rose et al., 2005) and are associated with lower academic achievement and behavioural problems, which may lead to worse functional outcomes in adulthood (Thuy et al., 2011). Moreover, performance on recognition memory tasks has been shown to be poorer in preterm infants compared with controls, in both auditory (Therien et al., 2004) and visual modalities (Rose et al., 2001).

The medial temporal lobe—the hippocampi and surrounding perihinal, entorhinal, and parahippocampal cortices—has been identified as a key brain area for visual recognition memory (Wixted and Squire, 2010). Volume reductions in the hippocampi are often described in preterm children (Peterson et al., 2000), adolescents (Nosarti et al., 2002), and young adults compared with full‐term controls (Aanes et al., 2015) and these have been associated with poorer performance on memory tasks (Aanes et al., 2015; Isaacs et al., 2000).

The fornix and the cingulum are two white matter tracts that connect the medial temporal lobe to other regions of the cortex. The fornix is a C‐shaped bundle that connects the hippocampi to the mammillary bodies and hypothalamus. The cingulum connects the medial temporal, posterior cingulate, and prefrontal cortices. Both tracts have been shown to be altered in terms of microstructure and volume in preterm young adults compared with term born controls (Eikenes et al., 2011; Nosarti et al., 2014; Salvan et al., 2014). In one study, smaller white matter volume in an area encompassing the fornix was associated with nonverbal memory only in very preterm individuals and not in controls (Nosarti et al., 2014).

To date, some work has been conducted to investigate the neural substrates of specific aspects of memory in very preterm adolescents and young adults using functional MRI (fMRI). Differential activation patterns have been found in very preterm samples compared to controls during visual (Brittain et al., 2014; Narberhaus et al., 2009) and verbal paired associate learning tasks (Kalpakidou et al., 2012; Lawrence et al., 2010; Salvan et al., 2014). In terms of structural‐functional relationships, Salvan et al. (2014) found decreased activation in a cluster encompassing part of the left hippocampus, thalamus and parahippocampal region in very preterm young adults compared with controls, as well as microstructural white matter alterations (e.g., lower fractional anisotropy values) in the very preterm group in pathways linking these regions, including the fornix. However, these findings were presented independently from one another and no specific associations between neural activation and task‐related tracts were reported. Froudist‐Walsh et al. (2015) used fMRI during performance of a working memory task to show a significant correlation between smaller dorsal cingulum volume and increased neural activation in bilateral perisylvian cortex, which is an area outside the typical working memory network. Increased perisylvian activation was only observed in very preterm adults who sustained perinatal brain injury and was interpreted as being compensatory, as it was positively correlated with task performance.

This study aimed to probe the long‐term consequences of very preterm birth on the functional and structural brain correlates of recognition memory, specifically investigating the relationships between: neural activation during completion of a visual recognition memory task that preferentially engages the hippocampi (adapted from Dove et al., 2006), volume of the hippocampi, and volume and microstructural attributes [hindrance modulated orientational anisotropy (HMOA)] of white matter tracts that connect to the hippocampi.

Based on the evidence assigning a critical role to the hippocampi in recognition memory processing, and reporting structural hippocampal alterations following very preterm birth, we predicted that adults born very preterm would show decreased neural activation in hippocampi during on‐line task performance. In addition, based on results of our recent multimodal neuroimaging study of working memory using the same cohort, which found significant associations in very preterm born adults between neural activation and volume of task‐specific white matter tracts (Froudist‐Walsh et al., 2015), we predicted that differential neural responses in the preterm group would be associated with white matter alterations of the fornix and the cingulum. Finally, we expected white matter connectivity to underlie the changes of local brain activations and volumetric characteristics.

## METHODS AND MATERIALS

Fifty‐two participants were recruited for the fMRI study from a cohort of individuals born very preterm admitted to the neonatal unit at University College Hospital, London, between 1979 and 1985. Fifty term born controls were also studied. Exclusion criteria for the control group included birth complications (e.g., low birth weight defined as <2500 g, endotracheal mechanical ventilation), prolonged gestation (greater than 42 weeks), history of psychiatric illness, severe hearing and motor impairments, and mental retardation indicated by IQ < 70. Participants gave full informed consent and the experiment was approved by the appropriate local ethics committees and in compliance with national legislation and the code of ethical principles for Medical Research Involving Human Subjects of the World Medical Association (Declaration of Helsinki).

### Recognition Memory Task

**Figure 1:**
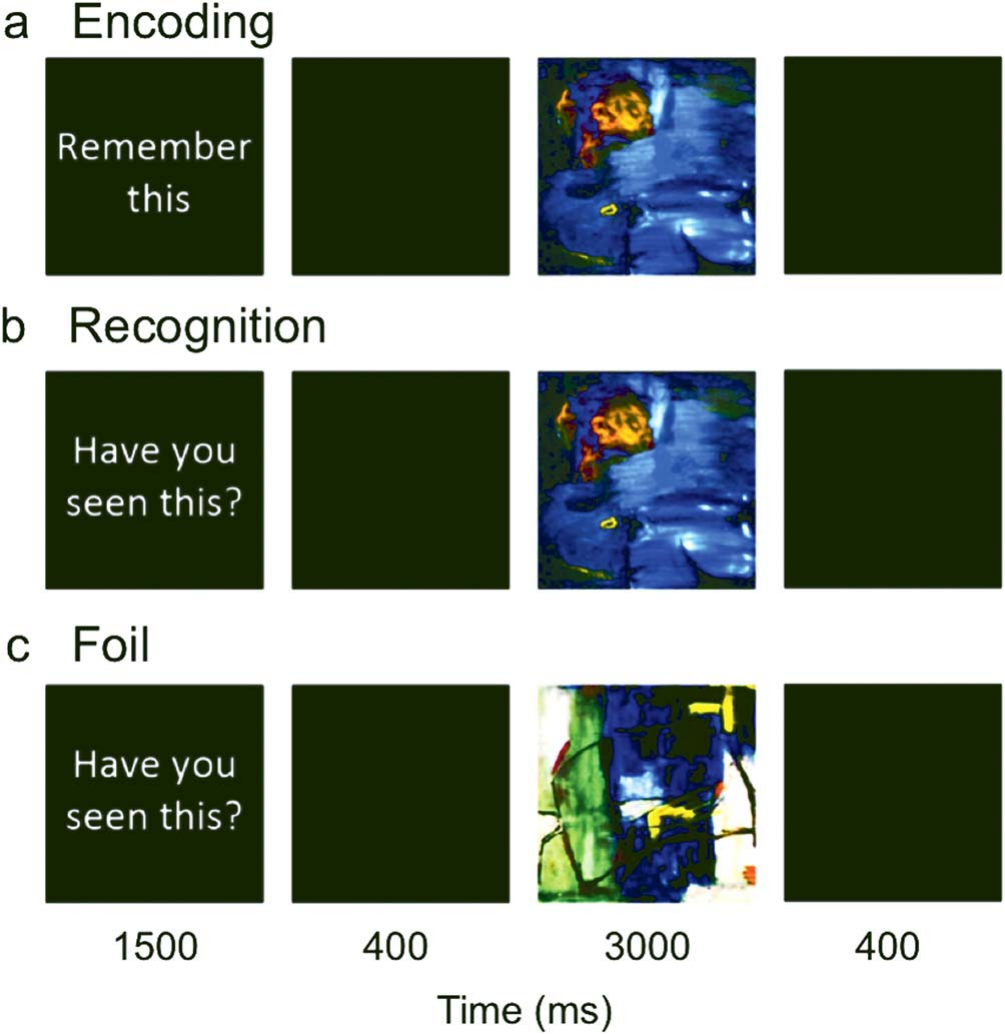
The visual recognition fMRI task paradigm. [Color figure can be viewed at http://wileyonlinelibrary.com.]

The aim of this experiment was to investigate the neural networks that are recruited during a recognition memory task by adults who were born very preterm. We adapted a task designed by Dove et al. (2006) which elicited robust activation in neural regions associated with recognition memory. There were three conditions: (1) encoding, (2) recognition, and (3) foil. During encoding trials, participants were instructed to remember an abstract art stimulus. During recognition trials, participants were presented with a previously seen abstract art image and had to indicate whether they recognised the image. During foil trials, participants were presented with a previously unseen image and asked to indicate whether they remembered the image. All trials were presented in an event‐related design and started with an instruction presented for 1.5 s. In encoding trials the instruction was “remember this,” in recognition and foil trials the instruction was “have you seen this?” A blank screen was then presented for 0.4 s followed by an abstract art stimulus presented for 3 s. Trials ended with a 0.4 s blank screen (Fig. 1). Participants responded using a two‐button MR‐compatible button box (made in house). The experiment consisted of 70 trials, 30 encoding, 30 recognition, and 10 foil trials; these trials were randomly interspersed. Thirty‐five nonevent, low level baseline trials (blank screen presented for 5.3 s) were intermixed throughout the experiment. Corresponding encoding and recognition trials were on average 67.8 s apart. The experiment lasted a total of 9.3 minutes. During encoding trials participants were told to press both buttons simultaneously after the image was presented. During recognition and foil trials, a left button press indicated participants had previously seen the image and a right button press indicated they had not. Participants were familiarised with the task prior to the fMRI experiment in an off‐line training session in which they were asked to make responses to example trials using a different set of images.

### Image Acquisition

Data were collected using a GE 3 tesla Signa MR scanner (GE Healthcare, USA) at the Institute of Psychiatry/Maudsley Hospital, Kings College London. A gradient‐echo EPI sequence was used to collect data from 39 non‐contiguous slices of 3.5 mm thickness separated by a distance of 0.5 mm, and with in‐plane voxel resolution of 3.75 × 3.75 mm^2^. These were co‐registered with T1‐weighted anatomical image (TR/TE/TI: 7.1/2.8/450 ms, matrix: 256 × 256), allowing for 196 slices with no gap and an isotropic resolution of 1.1 × 1.1 × 1.1 mm^3^. Diffusion weighted images were acquired using a multislice spin echo EPI sequence, obtaining 60 contiguous near‐axial slice locations with isotropic (2.4 × 2.4 × 2.4 mm^3^) voxels (TE/TR = 104.5 ms/range 12–20 RR intervals). Maximum *b*‐value was 1300 s/mm^2^, with 32 diffusion‐weighted directions and 4 non‐diffusion weighted volumes. Peripheral cardiac gating was applied with an effective TR of 20/30 RR interval.

### Functional MRI Analysis

Statistical analysis of fMRI data was performed using FEAT (http://www.fmrib.ox.ac.uk/fsl). The three initial volumes were removed to minimize the effects of magnetic saturation. Motion correction was followed by spatial smoothing (Gaussian, FWHM 5 mm) and temporal high‐pass filtering (sigma = 50 s). Regressors for each condition in the general linear model were convolved with a gamma hemodynamic response function. For the encoding trials, only the trials that were subsequently successfully recognised in the recognition trials were modelled. For the recognition trials, only those in which participants gave a correct response were modelled separately from incorrect trials. Only correct recognition trials were modelled in the analyses. Individual participant data were then entered into a higher‐level analysis using a mixed effects design (FLAME, http://www.fmrib.ox.ac.uk/fsl) whole‐brain analysis. Neural responses from each individual were registered to a study specific T1‐weighted template, which is an average of 78 brain images from term born and preterm individuals (Froudist‐Walsh et al., 2015).

Three contrasts were studied: encoding > low‐level baseline, recognition > low level baseline, and recognition > encoding. Our main contrast of interest was comparison of activation in the recognition trials compared to the encoding trials of the task. Contrasts were thresholded at *z* = 2.3 (*P* < 0.05, corrected) and from the resulting cluster maps clusters of activity that significantly differed between groups were identified. No statistically significant results were found when comparing very preterm adults with brain injury, very preterm adults with normal ultrasound classification (grouped according to neonatal ultrasound classification, as described in detail in Froudist‐Walsh et al., 2015) and controls; therefore, we focused on comparisons between the whole very preterm group and controls. In addition to exploring between group differences in patterns of neural response, we also investigated whether these differences in response were associated with on‐line task performance. We assessed task performance by means of *A* [Eq. (1)], a nonparametric signal detection sensitivity measure that takes into account both hit rate (*H*) and false alarms (*F*) (Zhang and Mueller, 2005). This estimate avoids making distributional assumptions and uses the estimate of the average area of possible receiver operating characteristic curves that are constrained by *H* and *F*. *A* can also be used when *H* and *F* is equal or close to 0 or 1.

(1) A=34+H−F4−F1−H   if F≤0.5≤H34+H−F4−F4H       if F≤H<0.5 34+H−F4−1−H4(1−F)   if 0.5<F≤H

Formula used for calculating *A*. *H* = Hit rate, *F* = False alarm rate.

### Normalization

Each individual's functional data were registered to their structural scan using FSL's FLIRT (Jenkinson et al., 2002; Jenkinson and Smith, 2001) and the boundary‐based registration (Greve and Fischl, 2009) cost function. This technique extracts the surfaces from the T1‐weighted image, and then aligns the fMRI data to the T1‐weighted data by maximising the intensity gradient across tissue boundaries. This method has been shown to be more accurate and robust to signal inhomogeneities than traditional intrasubject registration algorithms (Greve and Fischl, 2009). Then, FSL‐FNIRT (Andersson et al., 2010) was used to normalize each individual's structural data to the study specific template.

### Hippocampi Volume

Freesurfer, a set of automated tools for reconstruction of the brain from its T1‐weighted MRI data, was used to measure the volume of the left and right hippocampi in each individual. Freesurfer's volume‐based stream is designed to preprocess MRI volumes and label subcortical tissue classes. The stream consists of five stages: affine registration with MNI305 space, initial volumetric labeling, correction of the B1 bias field, nonlinear volumetric alignment to the MNI305 atlas, and labeling of the volume (Fischl et al., 2002, 2004). The volume of the left and right hippocampi were calculated and summed. Intracranial volume of each participant was also calculated and used as a nuisance factor in all analyses of hippocampal volumes.

### Tractography

Preprocessing of diffusion MRI data followed the pipeline developed by Froudist‐Walsh et al. (2015). Brain extraction was performed on the diffusion‐weighted and b0 images using FSL's BET. Motion and eddy‐current corrections was done on the brain‐extracted data using ExploreDTI (Leemans et al., 2009). A constrained spherical deconvolution approach was chosen to differentiate multiple directions within one voxel (Tournier et al., 2004). It was shown by Wilkins and colleagues (Wilkins et al., 2015) that tractography using constrained spherical deconvolution outperforms tractography using other reconstruction methods when using data acquired with clinical *b*‐values. Constrained spherical deconvolution was performed using a damped version of the Richardson‐Lucy algorithm (Dell'acqua et al., 2010). Parameters were optimised to find the best possible balance between resolving multiple fibre orientations and minimising false‐positive fibre orientation distributions (FOD). The parameters used were: regularisation threshold *η* = 0.02, fibre response function = 2, algorithm iterations = 300, and regularisation parameter *v* = 20. Fibre orientation estimates were taken from the orientation of the peaks of the FOD profile. We used an absolute (equal to 4 times the amplitude of a spherical FOD obtained from a grey matter voxel) and a relative threshold (equal to 7% of the amplitude of the maximum amplitude of the FOD at that voxel) at each voxel to remove the general noise floor and surviving noise local maxima, respectively. Each FOD that survived the threshold were used as seeds to perform whole‐brain tractography. Fibre orientation streamlines were propagated using Euler integration with a step‐size of 1 mm. Propagation stopped if the angle between two successive steps exceeded 60°. Tractography reconstruction was performed using software written in Matlab (http://www.mathworks.co.uk/products/matlab/; Dell'Acqua et al., 2013).

White matter dissection of the fornix and cingulum was performed in native diffusion space in TrackVis (trackvis.org) using a two‐region method (Catani and Thiebaut de Schotten, 2008). The fornix was dissected in each hemisphere and its commissure was not included. The cingulum was divided into two segments, a dorsal segment that connects the posterior cingulate to the prefrontal cortex, and a ventral segment that connects the hippocampi to the posterior cingulate cortex (PCC). All tracts were dissected in both hemispheres. Artefactual/nonanatomical fibres were removed using manually drawn region‐of‐avoidances. White matter tracts were evaluated by HMOA, a tract‐specific characterization of white matter diffusion properties.

### Statistical Analysis

Statistical analysis was done in SPSS 21. The distribution of the data was tested for normality using a Shapiro‐Wilk test. Not all variables were normally distributed; therefore, group comparisons were performed using Mann‐Whitney U tests. Neural activation, fibre tract characteristics, volume of the hippocampi, and gestational age (GA) were used as independent variables in a multiple linear regression model to find the best predictors of episodic memory performance (*A* scores). Each multiple linear regression model was built first across the whole sample, then subsequently for separate groups. Multicollinearity was tested for all independent variables and all variables had a variance inflation factor < 3. Partial correlation was performed to explore the relationship between multiple correlated variables. All models and tests were corrected for multiple comparison using the Benjamini‐Hochberg procedure (Benjamini and Hochberg, 1995).

## RESULTS

Only participants who achieved an accuracy higher than 50% on the fMRI task were included in the analysis to ensure they were actively performing the task. Three very preterm born participants were thus excluded. The final sample studied included 49 very preterm participants and 50 term born controls (Table 1). The very preterm and control groups did not show significant differences in sex; very preterm individuals were slightly older than controls. The two groups did not significantly differ in socio‐economic status. At time of assessment, one very preterm participant and nine controls were students; six very preterm participants and three controls were unemployed. The preterm group performed worse than controls on the on‐line task, as measured by *A* scores (*P* = 0.021). There were no very preterm participants who had severe hearing and/or motor impairments or mental retardation indicated by IQ < 70. Four very preterm participants had a current psychiatric diagnosis.

**Table 1:** Neonatal variables, demographics, and visual recognition memory task performance

	**Preterm (*n* = 49)**	**Control (*n* = 50)**	**Test statistic**	***P*‐value**
**Age (mean ± SD)**	30 ± 2.1	29.38 ± 3.53	U = 1502.5	0.05
**Sex (M/F)**	27/22	20/30	Chi‐square = 2.263	0.096
**GA**	29.31 ± 2.2	–	–	–
**Socio‐economic status** a				
I–II (Professional and Intermediate)	26	25	Fisher's exact = 4.652	0.108
III (Skilled manual and Nonmanual)	11	12		
IV–V (Semiskilled and Unskilled manual)	5	0		
***A* scores**	0.92 ± 0.08	0.95 ± 0.05	*U* = 896	**0.021**

a Her Majesty's Stationary Office, 1991.

*A* scores: a nonparametric signal detection sensitivity measure that takes into account both hit rate and false alarms.

### fMRI Results

Age was added as a covariate in the fMRI analysis as it was close to being significantly different between groups. In terms of neural response, regions differentially activated between the groups in the encoding > low level baseline and the recognition > low level baseline contrasts are reported in Table 2.

**Table 2:** Differential activations between very preterm adults and controls in the encoding > low level baseline and recognition > low level baseline contrasts

Encoding > Low level baseline	Region	Peak MNI coordinate [*x*,*y*,*z*] (mm)	Cluster size (voxels)	*P*‐value
**Preterm > Control**	Left LOC, superior division	[−30,−82,40]	9370	**<0.001**
	Right AG	[48,−48,56]	7318	**<0.001**
	Left SFG	[−2,18,66]	5171	**<0.001**
	Left MFG	[−32,32,46]	4959	**<0.001**
	Right MFG	[40,32,40]	4364	**<0.001**
	Left Orbital Frontal Cortex	[−36,38, −4]	2911	**0.004**
	Left Postcentral Gyrus	[−36, −32,64]	2753	**0.006**
Recognition > Low level baseline				
**Preterm > Control**	Right LOC, superior division	[38, −82,32]	3358	**0.002**
	Left Frontal Pole	[−38,52,6]	3174	**0.003**

AG: angular gyrus; LOC: lateral occipital cortex; MFG: middle frontal gyrus; MTG: middle temporal gyrus; SFG: superior frontal gyrus.

**Figure 2:**
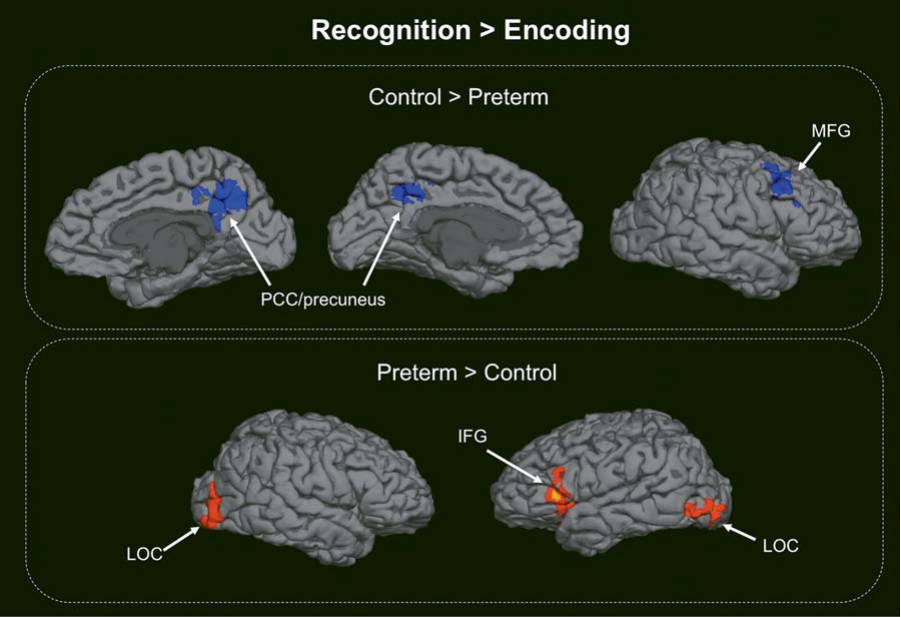
Between‐group differences in neural activation when contrasting recognition to encoding trials of the on‐line visual recognition task. PCC: posterior cingulate cortex; MFG: middle frontal gyrus; LOC: lateral occipital cortex; IFG: inferior frontal gyrus. [Color figure can be viewed at http://wileyonlinelibrary.com.]

When contrasting the recognition trials to the encoding trials, the very preterm group showed less activation in the PCC/precuneus and the right middle frontal gyrus (MFG) compared with controls. However, the very preterm group showed more activation in the left inferior frontal gyrus (IFG) and bilateral lateral occipital cortex (LOC) compared with controls (Table 3, Fig. 2).

**Table 3:** Differential activations between very preterm adults and controls when contrasting the recognition trials to the encoding trials

Recognition > Encoding	Region	Peak MNI coordinate [*x*,*y*,*z*] (mm)	Cluster size (voxels)	*P*‐value
**Control > Preterm**	PCC/precuneus	[2,−54,42]	6855	**<0.001**
	Right MFG	[48,26,44]	5339	**<0.001**
**Preterm > Control**	Right LOC, superior and inferior division	[40,−86,18]	5377	**<0.001**
	Left LOC, inferior division	[−46,−70,−6]	4295	**<0.001**
	Left IFG	[−56,14,−4]	3781	**0.002**

PCC: posterior cingulate cortex; MFG: middle frontal gyrus; LOC: lateral occipital cortex; IFG: inferior frontal gyrus.

### Structural MRI Results

**Figure 3:**
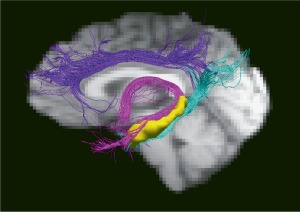
The dorsal cingulum is delineated in purple, the ventral cingulum in light blue, and the fornix in pink. The hippocampi are shown in yellow. [Color figure can be viewed at http://wileyonlinelibrary.com.]

Four participants were excluded from the analysis due to poor quality diffusion images (very preterm, *n* = 2; control, *n* = 1) and unsuccessful Freesurfer parcellation (very preterm, *n* = 1). The volume of the dorsal, ventral cingulum, and fornix were significantly different between the very preterm and control groups after controlling for intracranial volume. The HMOA of the white matter tracts was only significantly different between the groups in the fornix, with the very preterm group having lower HMOA than controls. Very preterm participants had smaller volumes of the hippocampi than controls, after adjusting for intracranial volume (Table 4, Fig. 3).

**Table 4:** White matter tract and hippocampal volume

		Preterm	Control	Test statistic	*P*‐value
Dorsal cingulum	Volume (ml)^a^	45.8 ± 15.31	51.83 ± 14.58	811	**0.009**
	HMOA	207.55 ± 27.84	216.19 ± 28.25	1014.5	0.191
Ventral cingulum	Volume (ml)^a^	16.29 ± 6.5	21.2 ± 9.08	808	**0.008**
	HMOA	162.41 ± 17.5	163.04 ± 18.7	1145.5	0.706
Fornix	Volume (ml)^a^	20.29 ± 10.25	28.28 ± 9.56	669	**<0.001**
	HMOA	164.07 ± 27.38	178.43 ± 24.94	808.5	**0.006**
Hippocampal volume (ml)^a^		8765 ± 1080.85	9306 ± 1301.57	593	**<0.001**

a Controlling for intracranial volume.

### Prediction of On‐Line Performance

**Figure 4:**
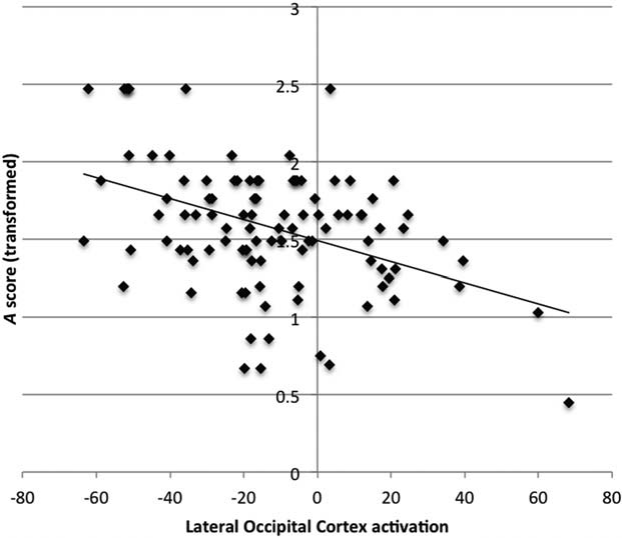
Scatterplot of *A* score and LOC activation in the whole sample.

All brain measures that were found to be significantly different between the groups were used as independent variables in a multiple linear regression model to predict *A* scores. *A* scores were transformed by ${\left(\sin^{-1}A\right)}^{2}$ to meet the assumptions of multiple linear regression. Regions with bilateral activation were averaged and used as one variable to avoid multicollinearity issues. Three multiple linear regression models were built using activation differences in encoding > low level baseline, recognition > low level baseline, and recognition > encoding contrasts. Structural brain variables were included in all models. Only the model built using activation differences in recognition > encoding was significant and LOC activation emerged as the best predictor of *A* scores (*P* < 0.001, adjusted *R* ^2^ = 0.204, Table 5, Fig. 4). Multiple linear regression models of the separate groups were not statistically significant.

**Table 5:** Coefficients of the multiple linear regression model to explain *A* scores in the whole sample

Independent variable	Standardised Coefficients (*β*)	*P*‐value
Right MFG	0.103	0.399
PCC/precuneus activation	0.216	0.074
LOC activation	−0.294	**0.020**
Left IFG	−0.119	0.324
Dorsal cingulum volume	0.063	0.548
Ventral cingulum volume	−0.095	0.391
Fornix volume	0.076	0.623
Fornix HMOA	−0.116	0.394
Hippocampal volume	−0.105	0.352

MFG: middle frontal gyrus; PCC: posterior cingulate cortex; LOC: lateral occipital cortex; IFG: inferior frontal gyrus; HMOA: hindrance‐modulated orientational anisotropy.

As recognition performance relies to a degree on the effectiveness of the previous encoding stage, we extracted LOC activation in the same location of the significant recognition > encoding contrast (LOC recognition, inferior division) in the encoding > low level baseline (LOC encoding, inferior division) contrast. We then built a multiple linear regression model including LOC recognition, inferior division, LOC encoding, inferior division, white matter structures, and volume of the hippocampi. In the whole sample, this model was significant (*P* < 0.001, adjusted *R* ^2^ = 0.195). However, only LOC recognition, inferior division was significant in explaining on‐line task performance, not LOC encoding in the same cluster. This result shows that while accounting for LOC activation during encoding, it is activation of the recognition > encoding contrast that significantly explains on‐line task performance.

### Functional‐Structural Relationships

To further explore the relationship between neural activation and local structure, subsequent multiple linear regression models were built to find variables that explained LOC activation. In the whole sample, this model was not significant (*P* = 0.077). However, when investigating the model separately for the very preterm and control groups, LOC activation was predicted by fornix HMOA in the very preterm group (*P* = 0.013, adjusted *R* ^2^ = 0.208, Table 6); this was not the case in controls (*P* = 0.901).

**Table 6:** Coefficients of the multiple linear regression model to predict LOC activation in the very preterm group

Independent variable	Standardised Coefficients (*β*)	*P*‐value
Dorsal cingulum volume	−0.273	0.065
Ventral cingulum volume	−0.194	0.212
Fornix volume	0.3	0.184
Fornix HMOA	−0.479	**0.020**
Hippocampal volume	−0.023	0.886

HMOA: hindrance‐modulated orientational anisotropy.

Both on‐line performance and fornix HMOA were found to be associated with LOC activation; therefore, we used a partial correlation to assess if fornix HMOA influenced the association between on‐line performance and LOC activation. The correlation between on‐line performance and LOC activation persisted after partialling out fornix HMOA in the whole sample (*r* = −0.421, *P* < 0.001). The same result was found in the control group (*r* = −0.451, *P* = 0.001), but not in the very preterm group (*r* = −0.308, *P* = 0.037 (not significant after multiple comparison correction).

After controlling for current psychiatric diagnosis in the very preterm group, results remained unaltered.

## DISCUSSION

This study demonstrated functional and structural brain alterations in very preterm born adults and a significant association between altered functional activation and recognition memory performance. These findings suggest the use of a suboptimal strategy to perform a recognition memory task in very preterm adults.

We found that preterm adults had decreased neural activity in the PCC/precuneus and right MFG when contrasting the recognition trial to the encoding trial, while increased activity was seen in the left IFG and bilateral LOC. Contrary to our hypothesis, we did not find decreased activation in the hippocampi in very preterm adults during a recognition memory task, despite them having reduced bilateral hippocampal volume compared to controls. Instead, less activation was observed in the PCC/precuneus. The PCC has been identified as a key node of the posteromedial memory system and is involved in the successful retrieval of information (Buckner et al., 2005). It is suggested that increased activation in the PCC during successful retrieval would reflect the ability to direct the attention to internal representations of the encoded memory, possibly through PCC‐medial temporal lobe connections (Wagner et al., 2005). In cognitively intact older individuals, hippocampal‐PCC intrinsic connectivity positively correlates with performance on a recognition memory task (Wang et al., 2010). Although this finding refers to samples undergoing neurodegenerative processes, it shows that the communication between the hippocampus and the posteromedial cortex is important for recognition memory performance.

Different patterns of neural activation were further observed in frontal lobe regions, with decreased activation in the right MFG and increased activation in the left IFG in the preterm group compared to controls. Involvement of the frontal cortex during recognition memory has been reported in the literature (Bar et al., 2006). Furthermore, a hemispheric encoding/retrieval asymmetry (HERA) model has been described, claiming a left prefrontal cortex specialization for encoding, and a right prefrontal cortex specialization for retrieval (Habib et al., 2003). From our results, we see a less enhanced HERA pattern in the very preterm group compared with controls. Previous studies have shown reductions in corpus callosum surface area in preterm infants and adolescents (Nosarti et al., 2004; Thompson et al., 2011), which may have affected the development of intrahemisphere specialisation in very preterm individuals.

Increased neural activation in bilateral LOC, an area typically involved in object perception and recognition, was associated with worse task performance. Lopez‐Aranda et al. discovered that the elimination of neurons in layer 6 of V2 visual cortex with no damage to the hippocampus resulted in a complete loss of object recognition memory (Lopez‐Aranda, et al., 2009). Their results support the view that the entire stream of ventral visual‐to‐hippocampus is important for visual memory processing, not only the medial temporal lobe. One hypothesis could be that very preterm adults required increased engagement of the LOC during an abstract art recognition memory task involving visual memory.

Regarding between‐group structural differences in memory related white matter tracts, the very preterm group had smaller volume of all tracts we considered (i.e., dorsal and ventral cingulum and fornix) compared to controls. However, significant differences in white matter microstructure in the very preterm group was only found in the fornix (lower HMOA values). Contrary to the present findings, in our recent study using the same cohort, Froudist‐Walsh et al. (2015) found that dorsal cingulum HMOA was reduced in very preterm individuals, although only in those who had sustained neonatal brain injury compared to very preterm individuals with no perinatal brain injury and controls. The same study found that dorsal cingulum volume was correlated with compensatory neural activation during a working memory task only in very preterm adults who sustained perinatal brain injury, arguing for the specific involvement of this tract in working memory processing. Our results suggest instead that the fornix may be selectively involved in recognition memory processing in very preterm adults, based on findings of a significant association between greater LOC activation and lower fornix HMOA in the very preterm group but not in controls. The fornix is part of the Papez circuit, which is involved in information transfer from short‐term memory to long‐term memory. In the Papez circuit, the fornix projects from the hippocampal formation to the mammillary bodies, then, the mammillothalamic tract connects the mammillary bodies to the anterior nucleus of the thalamus, which in turn projects through the thalamo‐cortical radiations to the cingulate gyrus. This loop is completed by the cingulum projecting back to the hippocampal formation. Damage to the fornix impairs the process of consolidation of memory and results in the inability to form new declarative memories. Alterations of this circuit may also explain the increased LOC effort in very preterm adults observed here.

Our findings of increased neural activation in right MFG and PCC in the control compared with the very preterm group are in support of the predictive coding hypothesis, a stimulus recognition scheme using an empirical Bayesian approach (Friston, 2005). This theory suggests that higher‐level cortical areas send predictions to lower‐level sensory areas based on past experience. The predictions are then compared with the sensory input and the error between the two is sent back to the higher‐level cortical areas. As predictions improve, a weaker prediction‐error BOLD signal is seen in the sensory cortex. Our results are consistent with the idea that controls may be making greater use of higher‐order areas, such as the right MFG and PCC, to achieve accurate sensory predictions, resulting in reduced prediction‐error signal in the LOC. This process is reduced in the very preterm sample, possibly due to weaker or less accurate predictions, perhaps as a result of structural damage to core components of recognition memory, namely the hippocampi and fornix.

Strengths of this study include the use of multiple MRI modalities to assess both functional and structural aspects of recognition memory. The HMOA measure we used to assess white matter structure can provide a tract‐specific diffusion measure, even in the presence of multiple fibres crossing in a single voxel, unlike conventional measures such as fractional anisotropy. This gives us higher sensitivity to detect white matter alterations and a more accurate representation of its microstructure.

This study complements and extends our previous work, which focused on working memory (Froudist‐Walsh et al., 2015), to assess different types of memory. It has been suggested that working memory may be a more promising target for cognitive training than recognition memory in very preterm individuals, as core working memory regions are not fully developed until adulthood and have more potential for compensatory functional adaptation, whereas core recognition memory regions reach maturity during childhood, therefore recognition memory impairments may be permanent and less amenable to improvement (Nosarti and Froudist‐Walsh, 2016). A recent finding in preterm born adults also supports this view, where intrinsic connectivity changes in visual and dorsal attention networks compensate for visual short‐term memory storage capacity deficits (Finke et al., 2015).

A limitation of this study is we did not have a large enough number of foil trials to test the differences between recognition and foil trials. Every participant achieved a higher than chance accuracy in recognising previous images, but when also considering correct rejections of new images in *A* scores, the very preterm group performed significantly worse than controls, suggesting they were less able to correctly reject novel images. Furthermore, we did not assess visual acuity, which can be affected in very preterm born adults (Birch and O'Connor, 2001) and may affect visual recognition performance. However, participants who wore prescription lenses were provided with MR compatible glasses matching their prescription while completing the memory task in the scanner.

A further limitation of the study refers to the fact that in our analyses we considered bilateral hippocampal volume, despite evidence of hemispheric specialization of the hippocampi, whereby the left hippocampus is involved in the processing of verbal material and the right hippocampus in the elaboration of nonverbal stimuli (Kennepohl et al., 2007). However, in this study no between group differences in neural activation were found in the hippocampi of either hemisphere, thus further analyses were performed using bilateral hippocampal volume. Future work is needed to study the different roles of the left and right hippocampi in very preterm individuals.

## CONCLUSION

This study was the first to investigate multimodal imaging correlates of visual recognition memory in very preterm adults, providing a unique opportunity to investigate neural plasticity in a neurodevelopmental framework. Very preterm born adults displayed different neuronal activation patterns during completion of a visual recognition memory task compared to controls. Specifically, increased activation in the LOC in the very preterm group was associated with worse task performance, which may reflect weaker sensory prediction, and with structural integrity of memory‐related white matter tracts. These findings are inconsistent with our previous study, which indicated that increased activation in a perisylvian region was associated with functional adaptation to successfully perform a working memory task (Froudist‐Walsh et al., 2015). These contrasting results may suggest that altered patterns of activation may not always be compensatory, but they may also be maladaptive (Turkeltauba et al., 2012). Further studies are required to determine whether these inconsistencies indicate that adaptive changes are both task and region specific and to examine how they may influence behaviour.
